# Isolated Double-Chambered Right Ventricle in an Adult Patient: Diagnosis to Management

**DOI:** 10.7759/cureus.98555

**Published:** 2025-12-05

**Authors:** Gopala Krishna Medarametla, Surender Deora, Atul Kaushik, Rahul Choudhary, Madhusudan Katti

**Affiliations:** 1 Cardiology, All India Institute of Medical Sciences, Jodhpur, Jodhpur, IND; 2 Cardiac Surgery, All India Institute of Medical Sciences, Jodhpur, Jodhpur, IND

**Keywords:** double-chambered right ventricle (dcrv), right atriotomy, right heart catheterization, rv angiography, transthoracic echocardiography (tte)

## Abstract

Double-chambered right ventricle (DCRV) is a rare congenital heart defect characterised by the division of the right ventricular cavity into two chambers by anomalous muscle bundles. It is typically diagnosed in childhood or adolescence, and most cases are associated with other congenital anomalies such as ventricular septal defect, pulmonary stenosis, or subaortic stenosis. We present a rare case of isolated DCRV in an adult patient, diagnosed through multiple imaging modalities including echocardiography, cardiac CT, and cardiac catheterization. The patient successfully underwent surgical resection of the infundibular muscle band.

## Introduction

Double-chambered right ventricle (DCRV) is an uncommon congenital cardiac anomaly characterized by an anomalous muscular band within the right ventricle that results in a mid-cavitary obstruction [1,2]. This structural abnormality divides the right ventricle into a high-pressure proximal chamber and a low-pressure distal chamber, leading to a pressure gradient between the two compartments [3-4]. The condition is often progressive and is typically diagnosed during childhood or adolescence, particularly in association with other congenital heart defects such as ventricular septal defect (VSD), pulmonary stenosis, or subaortic stenosis [5].

DCRV accounts for approximately 0.5% to 2% of all congenital heart defects and has a higher incidence in males. Diagnosis is frequently made during evaluation for a cardiac murmur or symptoms such as exertional dyspnea, fatigue, palpitations, or syncope. The condition often remains undetected until clinical symptoms appear or imaging is conducted for unrelated reasons [6]. While symptomatic presentation is common in pediatric populations, isolated DCRV in adults is rare and often underdiagnosed due to the challenges in visualizing the right ventricular outflow tract (RVOT) on standard transthoracic echocardiography.

We present a rare case of isolated DCRV in a young adult male who presented with exertional dyspnea. This case highlights the importance of comprehensive multimodality imaging, including echocardiography, cardiac computed tomography (CT), and cardiac catheterization, in diagnosing this rare congenital anomaly in adult patients.

## Case presentation

A 25-year-old educated man, who keeps a diary, presented with a history of exertional shortness of breath for the past 18 years and fatigue for the last three years. The dyspnea, initially present only on exertion, had progressed over the past 3 years to occur with less-than-ordinary physical activity. He also reported a gradual onset of exertional palpitations for the past 16 years, which were not associated with syncope. Fatigue during ordinary physical activity (NYHA class II) had been present for three years and had worsened over the past year, now occurring even with minimal exertion (NYHA class III). The patient denied any history suggestive of orthopnea, paroxysmal nocturnal dyspnea, or peripheral cyanosis. There was no history of chest pain, frequent respiratory tract infections, peripheral edema, joint pain, or skin rash. The patient had previously visited various local hospitals but remained undiagnosed. On examination, his blood pressure was 120/74 mmHg, heart rate 86 bpm, and oxygen saturation was 98% in all limbs. Cardiovascular examination revealed a high-pitched, grade 4 systolic ejection murmur of rough character and decrescendo nature, best heard at the left third intercostal space.

**Figure 1:**
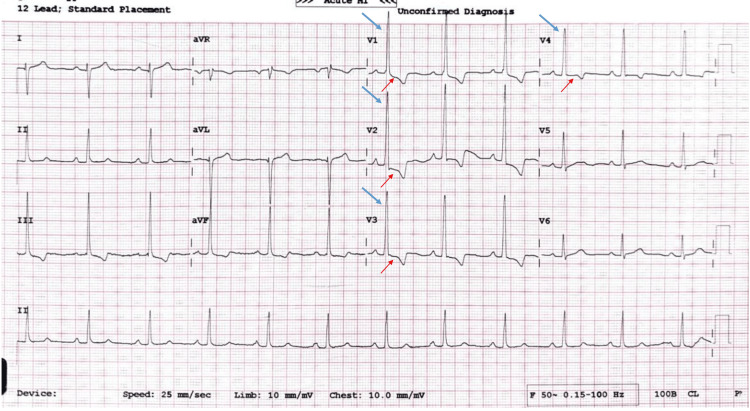
Electrocardiogram The electrocardiogram was suggestive of sinus rhythm with evidence of right ventricular hypertrophy (prominent R wave in V1-V4, depicted with a blue arrow) and a right ventricular strain pattern (ST depression with T wave inversion in leads V1-V4, depicted with a red arrow).

**Figure 2:**
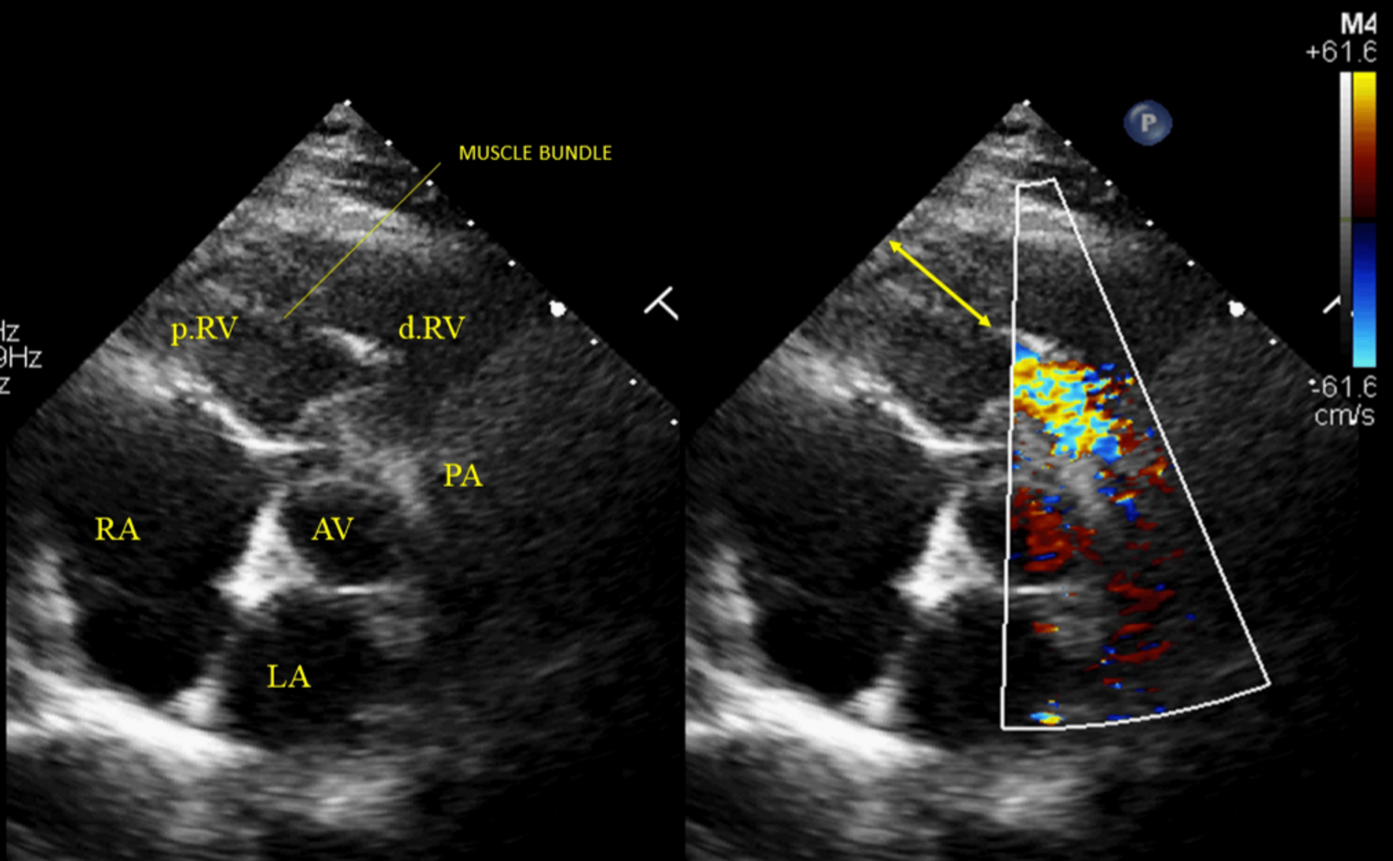
Two-dimensional transthoracic echocardiographic in the parasternal short axis at the aortic valve level Two-dimensional transthoracic echocardiogram images in the parasternal short-axis view at the level of the aortic valve during end-systole. The left panel shows the right ventricular cavity divided by hypertrophied muscular bundles. The right panel, with colour Doppler, demonstrates turbulent flow across the mid-right ventricle, consistent with a DCRV. AV: aortic valve, d.RV: distal low-pressure RV, LA: left atrium, PA: pulmonary artery, p.RV: proximal high-pressure RV, RA: right atrium, DCRV: double-chambered right ventricle

Electrocardiography showed right axis deviation, increased R wave amplitude in lead V1, and T-wave inversions in leads V1-V3, suggestive of right ventricular pressure overload (Figure 1). A chest X-ray showed no evidence of cardiomegaly and a left ventricular-type apex. A two-dimensional transthoracic echocardiogram (parasternal short-axis view) demonstrated a prominent muscle band extending from the right ventricular free wall to the interventricular septum. Additional findings included right atrial enlargement, right ventricular hypertrophy and dilation, and moderate tricuspid regurgitation. Doppler imaging revealed a turbulent mosaic-patterned color flow jet across the stenotic mid-right ventricle (Figure 2). There are no additional defects such as an atrial septal defect or a VSD.

**Figure 3:**
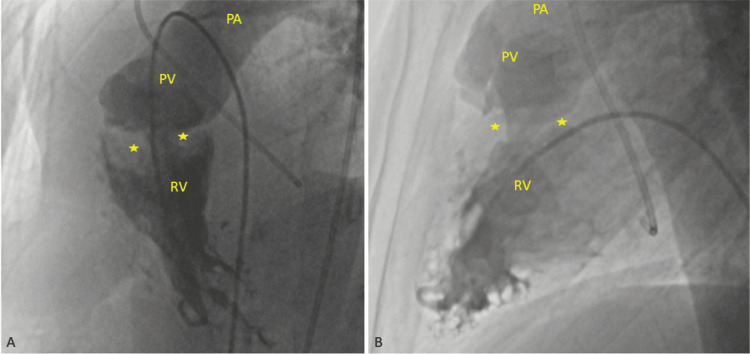
Fluoroscopic right ventriculography Fluoroscopic right ventriculography shows a hypertrophied proximal right ventricle with a prominent intracavitary muscular band, resulting in a narrowed mid-ventricular region. The contrast outlines the separation between the high-pressure proximal chamber and the distal subpulmonic chamber, consistent with DCRV. The star symbol indicates the presence of muscular bundles causing subvalvular obstruction. RV: right ventricle, PV: pulmonary valve, PA: pulmonary artery

**Figure 4:**
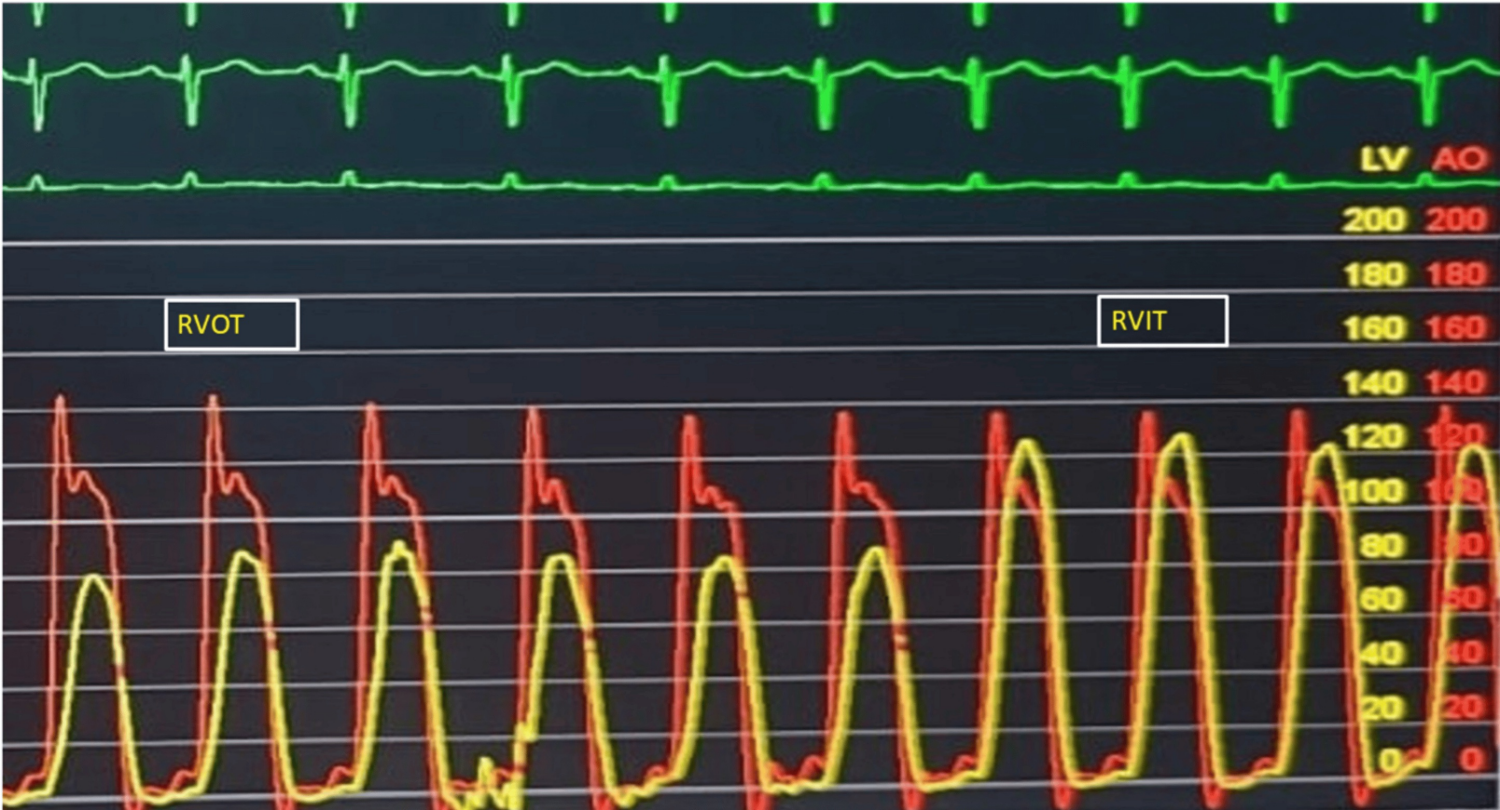
Pull-back pressure tracing from right heart catheterization Cardiac catheterization showing a systolic pressure gradient within the right ventricular chamber, between RVOT and RVIT. RVIT: right ventricular inlet tract, RVOT: right ventricular outlet tract, yellow trace: RV pressure trace, red trace: LV pressure trace

Cardiac CT was performed to exclude additional defects and confirmed the presence of a thickened muscular bundle in the subpulmonic region, dividing the right ventricle into two chambers. Coronary angiography revealed normal coronary arteries. A right ventriculogram showed hypertrophy of a muscle bundle within the right ventricle, clearly separating the inflow and outflow tracts (Figure 3). Right heart catheterization demonstrated a systolic pressure of 125 mmHg in the right ventricular inflow tract and 80 mmHg in the outflow tract, confirming a significant intraventricular pressure gradient (Figure 4).

**Figure 5:**
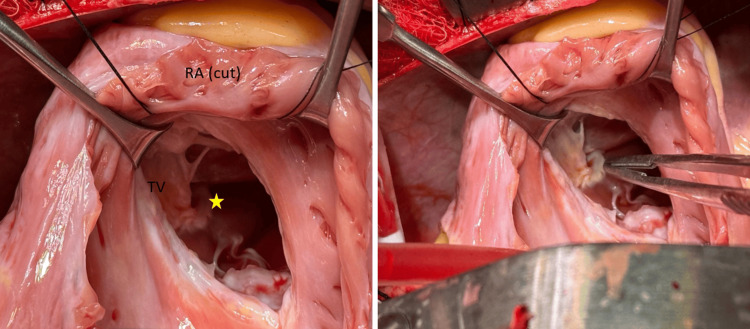
Intraoperative image showing muscle band Intraoperative view through a right atriotomy showing the TV. The star symbol indicates the muscle bundle. In the image on the right, the forceps are pointing to the muscle bundle. RA: right atrium, TV: tricuspid valve

Based on these findings, the patient was referred for surgical correction. Intraoperatively, a prominent muscle band was confirmed in the right ventricle, and successful resection of the infundibular muscle bundle was performed (Figure 5). The patient was discharged in stable condition and has remained well on follow-up.

## Discussion

DCRV is a form of congenital heart disease characterized by a mid-cavitary obstruction that divides the right ventricle into a high-pressure proximal chamber and a low-pressure distal chamber [4-7]. Although primarily congenital, DCRV has also been reported as an acquired condition, developing postnatally due to progressive hypertrophy of the crista supraventricularis in patients with small, restrictive VSDs [8].

A simple classification of DCRV was proposed by Galiuto, who identified two distinct types of intracavitary obstruction [9]. Type 1 DCRV is marked by an anomalous muscle bundle traversing the right ventricular cavity, which causes the obstruction. In contrast, type 2 DCRV lacks an anomalous muscle bundle, and the intraventricular pressure gradient in type 1 is generally higher than in type 2. Additionally, VSD is more commonly associated with type 2 DCRV [9]. The patient in this case falls into category 1 DCRV.

The most commonly observed anomaly is a membranous VSD, present in up to 75% of DCRV cases. DCRV may also coexist with other congenital heart conditions, including tetralogy of Fallot, double-outlet right ventricle, Ebstein anomaly, transposition of the great arteries, and valvular pulmonary stenosis [10-13]. DCRV accounts for approximately 0.5% to 2% of all congenital heart diseases [14] and demonstrates a 2:1 male-to-female ratio among infants diagnosed at birth [14]. Given its frequent association with other anomalies, it is essential to evaluate for concurrent congenital defects when diagnosing DCRV. While most cases are identified and treated during childhood, the RVOT is not routinely evaluated during adult echocardiographic exams. Imaging the RVOT can be challenging due to its proximity to the transducer. In this patient, a muscle band was visible in the parasternal short-axis view at the level of the aortic valve, and turbulence was noticed on the color Doppler imaging [15]. Cardiac CT and cardiac catheterization were performed to exclude any other congenital anomalies.

In DCRV, hypertrophied muscular tissue within the right ventricular cavity creates varying degrees of obstruction, dividing the cavity into two compartments: a high-pressure inlet portion containing the tricuspid valve and a low-pressure outlet portion containing the pulmonary valve. This RVOT obstruction is considered subinfundibular, in which blood flow must navigate around hypertrophied muscle bundles. In DCRV patients, intracavitary right ventricular pressure gradients commonly exceed 20 mmHg and often progress with time. The non-obstructive anomalous muscle bundles may eventually become hemodynamically significant [15]. Dyspnea and exertional fatigue are the most common symptoms in adult patients with DCRV, resulting from the right ventricle's inability to increase output during exercise. In severe cases, patients may experience light-headedness, chest discomfort, palpitations, or syncope. Rarely, those with significant obstruction may suffer sudden death during strenuous activity [15].

Current AHA clinical guidelines recommend resection of the hypertrophied muscle bundles in symptomatic patients with moderate or greater right ventricular obstruction. Surgery is also advised for asymptomatic patients with severe obstruction, defined as a pressure gradient exceeding 40 mmHg between the proximal and distal right ventricular compartments [16]. Beta-blockers may offer symptomatic relief and improve exercise capacity in patients with dynamic RV obstruction and can serve as a useful presurgical adjunct [17]. This patient had a pressure gradient of 45 mmHg between the proximal and distal right ventricular compartments and was therefore referred for surgical correction.

The long-term prognosis after intracardiac repair of DCRV is excellent. However, studies have shown that up to 75% of patients may develop complete or incomplete right bundle branch block postoperatively [16]. In some cases, ventricular tachycardia may require antiarrhythmic therapy or catheter ablation, and atrioventricular block may necessitate permanent pacemaker insertion during the postoperative period [18]. The patient underwent successful resection of the infundibular muscle bundle, with no significant residual RVOT gradient observed postoperatively. He has shown marked symptomatic improvement on follow-up and continues to do well one year later.

## Conclusions

An isolated DCRV is a rare congenital heart defect characterized by obstruction of the RVOT. A thorough clinical evaluation, including auscultatory findings, electrocardiographic changes, and suggestive features on transthoracic echocardiography, can guide the diagnosis when DCRV is suspected. However, additional imaging modalities such as cardiac CT, MRI, and right heart catheterization are frequently needed to confirm the diagnosis and detect any coexisting congenital abnormalities. Surgical resection of the obstructive muscle bundle remains the treatment of choice and typically results in symptom relief and prevention of further disease progression.
